# Breastfeeding Duration and Residential Isolation amid Aboriginal Children in Western Australia

**DOI:** 10.3390/nu4122020

**Published:** 2012-12-13

**Authors:** Elizabeth A. S. Cromie, Carrington C. J. Shepherd, Stephen R. Zubrick, Wendy H. Oddy

**Affiliations:** 1 Centre for Population Health Research, Curtin University, Perth, WA 6845, Australia; E-Mails: elizabethcromie@gmail.com (E.A.S.C.); carringtons@ichr.uwa.edu.au (C.C.J.S.); 2 Telethon Institute for Child Health Research, Centre for Child Health Research, University of Western Australia, P.O. Box 855, Perth, WA 6009, Australia; E-Mail: steve@ichr.uwa.edu.au; 3 Faculty of Medicine, Dentistry and Health Sciences, University of Western Australia, Perth, WA 6009, Australia

**Keywords:** breastfeeding duration, Australian Aboriginal children, isolation

## Abstract

Objectives: To examine factors that impact on breastfeeding duration among Western Australian Aboriginal children. We hypothesised that Aboriginal children living in remote locations in Western Australia were breastfed for longer than those living in metropolitan locations. Methods: A population-based cross-sectional survey was conducted from 2000 to 2002 in urban, rural and remote settings across Western Australia. Cross-tabulations and multivariate logistic regression analyses were performed, using survey weights to produce unbiased estimates for the population of Aboriginal children. Data on demographic, maternal and infant characteristics were collected from 3932 Aboriginal birth mothers about their children aged 0–17 years (representing 22,100 Aboriginal children in Western Australia). Results: 71% of Aboriginal children were breastfed for three months or more. Accounting for other factors, there was a strong gradient for breastfeeding duration by remoteness, with Aboriginal children living in areas of moderate isolation being 3.2 times more likely to be breastfed for three months or more (*p* < 0.001) compared to children in metropolitan Perth. Those in areas of extreme isolation were 8.6 times more likely to be breastfed for three months or longer (*p* < 0.001). Conclusions: Greater residential isolation a protective factor linked to longer breastfeeding duration for Aboriginal children in our West Australian cohort.

## 1. Introduction

Current recommendations from leading global organisations such as the World Health Organisation (WHO) are that for the first six months of life infants should be exclusively breastfed [1]. Breast milk is the ideal nourishment for infants’ optimal survival, growth and development [2]. After six months, it is recommended that breastfeeding be complemented with the introduction of solids, and continued into the second year of life [1]. Global statistics indicate substantial differences between these recommendations and reality for a vast number of children [3,4]. Traditionally, Australian Aboriginal children (the term *Aboriginal people* is used in this paper to refer to Australian Aboriginal and Torres Strait Islander peoples) were exclusively breastfed for six months, continuing to breastfeed for at least two years [5]. However there is limited information on current breastfeeding practices in Australian Aboriginal groups, and the relative rates in remote to non-remote populations. In addition, there is conflicting evidence on the factors that affect breastfeeding duration among populations around the world [6] including Indigenous groups.

We hypothesise that Aboriginal children living in very remote locations in Western Australia are breastfed for a longer duration than Aboriginal children living in metropolitan locations, and that residential location impacts on other determinants of breastfeeding duration. This study used a robust survey of Aboriginal early development to examine the factors that impact on the duration of breastfeeding among Western Australian Aboriginal children.

## 2. Experimental Section

### 2.1. Population and Sampling

Data in this report were from the Western Australian Aboriginal Child Health Survey (WAACHS) [7]. The WAACHS is a population representative study of the health, development and education of Aboriginal children aged 0–17 years in the state of Western Australia, and their families and communities. The survey was conducted between May 2000 and June 2002. An area-based clustered multi-stage sample design was employed to select dwellings (the term *dwelling* refers to any place where people reside). From 166,290 dwellings in 761 census collection districts, 139,000 dwellings were approached to determine if residents were eligible to participate in the survey. Throughout urban, rural and remote areas of Western Australia, a random sample of 6209 eligible children from 2386 families was selected. Consent to participate in the survey was obtained from 84% (1999) of families, totalling 5513 eligible children. Information was gathered via face-to-face interviews with the primary carer (usually the mother). Useable data were returned on 96% (5289) of participating children. Full details of the survey methods including content, non-response analysis, and weighting are available elsewhere [7,8,9,10]. 

### 2.2. Estimation

Sample data were weighted to represent the total population of Aboriginal children in Western Australia, and these weighted estimates have been consistently reported throughout the manuscript to provide a sense of the population scale and impact. While the WAACHS sample was broadly representative of the population of Aboriginal children living in Western Australia, comparisons with population benchmarks indicated that age, household size and region were significantly associated with non-response. The sample had a lower proportional representation of older children and children living in small households and the south-west region of Western Australia (including the Perth metropolitan area). Post-stratification weighting was employed to adjust for differences in response rates by age, household size and region and produce unbiased estimates.

The weighted estimates are based on 3932 Aboriginal children aged 0–17 years whose primary caregiver is their birthmother, and who were not still being breastfed at the time of the survey. This represents 74% of the total WAACHS sample (3932/5289 = 74.3%). The remaining 25.7% included children still being breastfed at the time of the survey and those whose primary carer was not their birth mother (and therefore were not able to respond regarding the duration of breastfeeding). The study sample was restricted to birth mothers as they are the most reliable source of information on early infant feeding. The weighted population estimate of in-scope children derived from the survey sample is 22,100 (or 74.2%; 22,100/29,800) children. 

Standard errors for survey estimates of total numbers of children in various categories were produced using the Ultimate Cluster Variance estimation technique [11]. Standard errors for estimates of proportions, percentages and ratios were calculated using a modified form Jack knife variance estimation technique [12]. 

### 2.3. Ethics Approval

The WAACHS received approvals from institutional ethics committees for mainstream and Aboriginal research. Consent was obtained from carers to collect WAACHS data and also to link their survey records with birth and hospital admission records. All components of the WAACHS are accountable to the Western Australian Aboriginal Child Health Survey Steering Committee. This ensured appropriate Aboriginal supervision and ownership of the data and procedures employed. 

### 2.4. Outcome and Exposures

#### 2.4.1. Outcome

The main outcome for these analyses was breastfeeding duration. During the WAACHS data collection, information on infant feeding was collected in both the infant (ages 0–3 years) and child health questionnaires (4–17 years old) as binary and categorical variables: “Was your child breastfed?” (yes, no), “How long was the child breastfed?” (0 to <3, 3 to <6 months, 6 to <9 months, 9 to <12 months, 12 months or more and still being breastfed), “Is your child still being given only breast milk?” (yes, no), and, “For how long was your child given only breast milk?” (answer in months) [13,14]. Given that the age range of WAACHS children was 0–17 years, much of the breastfeeding information was collected retrospectively. For these analyses, the breastfeeding data were dichotomised into “breastfed for less than three months” and “breastfed for three months or longer”. 

#### 2.4.2. Exposures

The state of Western Australia (WA) covers over one third of the Australian continental landmass. More than two thirds of WA’s total population and one third of its Aboriginal population reside in the metropolitan area of Perth. South-western WA contains many small population groupings and large areas of forest and for agriculture. Some of the most sparsely populated and remote areas in the world are in the centre and northwest of WA [15]. 

The survey sample was designed to be largely representative of the urban, regional and remote circumstances in which Western Australian Aboriginal people lived. The key exposure of interest was geographic remoteness or isolation. An index called Level of Relative Isolation (LORI) was created specifically for use in the WAACHS. The rationale for LORI is that it allowed “greater discrimination of the circumstances of survey respondents with respect to their isolation from population centres of various sizes and better differentiation between areas and communities that are extremely remote from major metropolitan centres” [7]. The five levels of LORI are “None”, “Low”, “Moderate”, “High”, and “Extreme”, with “None” representing the Perth metropolitan area. The LORI has been found to be sensitive to culture and language variations as well as to proximity and access to a range of services [9]. Full details on its development and validation are available elsewhere [7].

In addition to the LORI, confounding measures that occurred around the time of breastfeeding were considered in cross-tabulations and multivariate logistic regression analyses. Already identified in the literature, these were family size, socio-economic disadvantage, infant gender and birth weight, maternal characteristics of smoking and chewing tobacco in pregnancy, primary caregiver level of education attainment, primary caregiver occupation skill level and primary caregiver response to questions about family financial strain. 

#### 2.4.3. Birth Linkage Data

By consent, birthweight data were obtained by means of record linkage of the survey participants to the Maternal and Child Health Research Data Base (MCHRDB). The MCHRDB comprises all birth information recorded by attending midwives in Western Australia, and was linked to WAACHS data by probabilistic record linkage techniques. About 12% of WAACHS subjects were not able to be linked to their birth records; these were predominantly instances where consent was not given or the children were born outside of Western Australia.

### 2.5. Analyses

Standard Chi Square tests and Chi-Square tests for trend were used to assess the differences between categorical breastfeeding outcomes and the exposures and confounding measures identified from the study. Significance was set at alpha = 0.05. The exposures and confounding measures tested in the bivariate analysis were level of relative isolation (LORI), number of children in the family, quintiles of socio economic disadvantage, infant gender and birth weight, and maternal characteristics of smoking in pregnancy, chewing tobacco in pregnancy, level of education attainment, occupation skill and family financial strain categories.

Exposures and confounding measures that showed significant or near significant associations (*i.e.*, 95% CI upper limit did not fall within the range of a related category or was only slightly within range) were included in a multivariate analysis where the dependent variable, duration of breastfeeding, used the modelling probability of breastfeeding for three months or more, with only significant associations kept in the multivariate models. SAS version 9 [16] was the statistical package used for all data analyses.

## 3. Results

Of the 22,100 Aboriginal children aged 0–17 years within the scope of this study, 51% were male, 9% had a birth-weight of less than 2500 g and 44% lived in a family with four or more children. In relation to LORI, 7% lived in areas of extreme isolation, 26% lived in areas of low isolation and 38% lived in areas of no isolation (Table 1). Table 1 also highlights a range of characteristics of the primary caregivers of these children—the birth mothers of 54% of children did not smoke during pregnancy, 97% did not chew tobacco during pregnancy, and 79% had completed ten years or more of education. The majority of Aboriginal children had been breastfed for three months or more (71.1%; 95% CI 68.9, 73.1) and 28.9% (95% CI 26.9, 31.1) were breastfed for less than three months.

**Table 1:** Demographic, maternal and infant characteristics of Western Australian Aboriginal children aged 0–17, whose primary caregiver is their birth mother (*n* = 22,100) *.

Exposures and confounding measures			%	*n*
*Demographic variables*				
	Level of Relative Isolation (LORI)			
		None	37.8	8360
		Low	25.7	5680
		Moderate	20.0	4420
		High	9.1	2010
		Extreme	7.4	1640
	Number of children in the household			
		Four or more children	43.9	9690
*Infant characteristics*				
	Gender			
		Male	51.1	11,300
		Female	48.9	10,800
	Birth weight ^†^			
		<2500 g	9.0	2000
		>2500 g	79.0	17,500
*Maternal characteristics*				
	Primary caregiver: attainment ^§^			
		Did not attend school	1.4	310
		1–9 years of education	17.8	3930
		10 years	46.7	10,300
		11–12 years	26.3	5800
		>13 years	5.9	1300
	Family financial strain ^║^			
		Spending more money than we get	8.5	1870
		We have just enough money to get by	45.2	10,000
		Some money left over but we just spend it	13.2	2910
		Can save a bit now and again	26.6	5880
		Can save a lot	4.5	1000
	Smoking in pregnancy			
		Yes	46.3	10,200
	Mother chewed tobacco during pregnancy			
		Yes	3.5	760
	Occupation skill level ^¶^			
		Managers, administrators, professionals	3.9	870
		Associate professionals	3.4	740
		Tradespersons, advanced clerical, service workers	2.5	540
		Intermediate production and transport workers	13.9	3070
		Elementary clerical, sales & services, labourers	8.2	1810
		Not in the labour force or unemployed	66.2	14,600
	Socioeconomic disadvantage			
		Bottom 5%	56.7	12,500
		>5%–10%	25.6	5650
		>10%–25%	13.1	2890
		>25%–50%	4.2	930
		Top 50%	0.4	100

* Using the weighted data 22,100/29,800 of Aboriginal children aged 0–17 years, whose primary caregivers were also their birth mothers, excluding those still being breastfed in the West Australian Aboriginal Child Health Survey. All numbers are rounded estimates. The frequencies of missing responses have not been reported. ^†^ The figures for birth weight have been derived by excluding the “not stated” category (12.0%). ^§^ The figures for primary caregiver levels of educational attainment have been derived by excluding the “not stated” category (2.0%). ^║^ The figures for Family financial strain (primary caregiver response) have been derived by excluding the “missing” category (2.0%). ^¶^ The figures for occupational skill level have been derived by excluding the “missing” category (2.0%).

### 3.1. Bivariate and Multivariate Analyses

**Figure 1:**
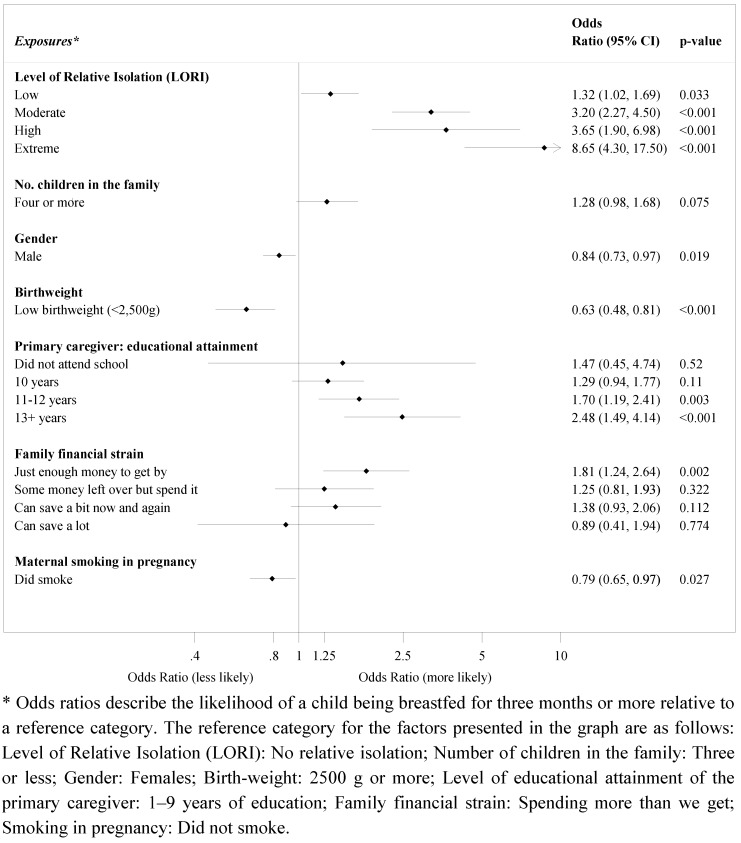
Multivariate relationship between location of residence and other demographic factors, infant and maternal characteristics with breastfeeding duration of three months or more, in Western Australian Aboriginal children aged 0–17, whose primary caregiver is their birth mother *.

Table 2 shows breastfeeding duration categorised into breastfeeding for less than 3 months and three months or more. The proportions in Table 2 are similar (and not significantly different) between males and females. Figure 1 shows the multivariate relationship between demographic factors.

**Table 2:** Bivariate analysis: Comparison of breastfeeding duration to demographic, maternal and infant characteristics of Western Australian Aboriginal children aged 0–17, whose primary caregiver is their birth mother *.

		Breastfeeding Never or <3 months (%)		Breastfeeding >3 months (%)	
Exposures		%	95% CI	%	95% CI
*Demographic characteristics*					
Levels of Relative Isolation (LORI)					
	None	40.6	36.5, 44.9	59.4	55.1, 63.5
	Low	33.4	29.2, 37.8	66.6	62.2, 70.8
	Moderate	16.0	12.9, 19.6	84.0	80.4, 87.1
	High	12.9	9.3, 17.5	87.1	82.5, 90.7
	Extreme	8.6	5.6, 13.0	91.4	87.0, 94.4
	Total	28.9	26.9, 31.1	71.1	68.9, 73.1
Number of children in the family—grouped					
	Three or less	34.2	30.9, 37.5	65.8	62.5, 69.1
	Four or more	22.2	19.7, 24.8	77.8	75.2, 80.3
	Total	28.9	26.9, 31.1	71.1	68.9, 73.1
*Infant characteristics*					
Gender					
	Male	30.3	27.5, 33.4	69.7	66.6, 72.5
	Female	27.5	24.9, 30.2	72.5	69.8, 75.1
	Total	28.9	26.9, 31.1	71.1	68.9, 73.1
Birth weight					
	Less than 2500 g	41.1	34.4, 48.6	58.9	51.4, 65.6
	2500 g or more	27.5	25.2, 29.8	72.5	70.2, 74.8
	Missing	29.3	23.6, 35.4	70.7	64.6, 76.4
	Total	28.9	26.9, 31.1	71.1	68.9, 73.1
*Maternal characteristics*					
Primary caregiver: level of educational attainment					
	Did not attend school	24.3	10.7, 44.9	75.7	55.1, 89.3
	1–9 years education	33.4	28.0, 39.5	66.6	60.5, 72.0
	10 years education	30.5	27.3, 33.7	69.5	66.3, 72.7
	11–12 years education	25.2	21.0, 29.6	74.8	70.4, 79.0
	>13 years education	24.5	15.3, 36.1	75.5	63.9, 84.7
	Not Stated	19.1	6.8, 40.7	80.9	59.3, 93.2
	Total	28.9	26.9, 31.1	71.1	68.9, 73.1
Family financial strain					
	Spending more money than we get	38.4	29.6, 47.9	61.6	52.1, 70.4
	We have just enough money to get by	27.6	24.7, 30.6	72.4	69.4, 75.3
	Some money left over but we just spend it	33.1	26.9, 40.3	66.9	59.7, 73.1
	Can save a bit now and again	27.3	23.0, 32.1	72.7	67.9, 77.0
	Can save a lot	26.3	17.6, 37.8	73.7	62.2, 82.4
Smoking in pregnancy					
	Yes	32.8	29.6, 36.2	67.2	63.8, 70.4
	No	25.6	22.9, 28.4	74.4	71.6, 77.1
	Total	28.9	26.9, 31.1	71.1	68.9, 73.1
Mother chewed tobacco during pregnancy					
	Yes	13.1	7.8, 20.1	86.9	79.9, 92.2
	No	29.5	27.4, 31.7	70.5	68.3, 72.6
	Total	28.9	26.9, 31.1	71.1	68.9, 73.1
Primary caregiver: occupation skill level					
	Managers, administrators, professionals	21.1	13.7, 31.2	78.9	69.4, 86.6
	Associate professionals	29.0	17.1, 43.1	71.0	56.9, 82.9
	Tradespersons, advanced clerical, service workers	22.9	9.0, 43.6	77.1	56.4, 91.0
	Intermediate production and transport workers	22.3	16.5, 29.0	77.7	71.0, 83.5
	Elementary clerical, sales & services, labourers	25.5	18.9, 32.6	74.5	67.4, 81.1
	Not in labour force or unemployed	31.7	29.1, 34.5	68.3	65.5, 70.9
	Missing	19.1	6.8, 40.7	80.9	59.3, 93.2
	Total	28.9	26.9, 31.1	71.1	68.9, 73.1
Quintiles of Socio-economic disadvantage					
	Bottom Quintile	27.2	24.7, 30.0	72.8	70.0, 75.3
	Second Quintile	31.5	26.6, 36.6	68.5	63.4, 73.4
	Third Quintile	28.9	22.5, 35.6	71.1	64.4, 77.5
	Fourth Quintile	36.2	19.4, 57.6	63.8	42.4, 80.6
	Top Quintile	26.7	0.8, 90.6	73.3	9.4, 99.2

* The table title is indicative of the weighted estimated population of Aboriginal Children aged 0–17 whose primary caregiver is the birth mother, excluding those still being breastfed in the West Australian Aboriginal Child Health Survey. (*n* = 22,100). All numbers are rounded estimates.

#### 3.1.1. LORI

The vast majority (91.4%; 95% CI 87.1, 94.4) of Aboriginal children living in areas of extreme isolation were breastfed for three months or more. There was a gradient effect across areas of isolation, with lower proportions being breastfed for three months or more in areas of moderate isolation (84.0%; 95% CI 80.4, 87.1) and the Perth metropolitan area (59.4%; 95% CI 55.1, 63.5).

This trend remained in multivariate logistic regression, where we showed that children in areas of moderate isolation were 3.2 times more likely to breastfeed for three months or more compared with those in Perth. These odds increased to 8.6 among Aboriginal children in areas of extreme isolation.

#### 3.1.2. Confounding Measures

Protective factors for longer breastfeeding duration included higher levels of maternal education, where multivariate analyses showed that Aboriginal children of mothers with 11–12 years and >13 years of education were 1.70, and 2.48, times more likely respectively to be breastfed for longer than children of mothers with 1–9 years of education.

Being a non-smoker during pregnancy was associated with breastfeeding duration, where multivariate analyses showed a protective relationship when children were less likely to be breastfed for three months or more if there was maternal smoking during pregnancy (*p* = 0.027). Other protective variables for longer breastfeeding duration included; having a birth weight of 2500 g or more as children with a lower birth weight were less likely to be breastfed for three months or more (OR 0.63, *p* ≤ 0.001); being female, where male children were slightly less likely to be breastfed for three months or longer compared with female (*p* = 0.019); and, being from families with four or more children, as they were 1.28 times more likely to be breastfed longer. 

Bivariate analyses of “Primary caregiver response to family financial strain” data showed a greater proportion of those in the lowest category of financial strain were less likely to breastfeed for three months or more when compared with the remainder of categories (38.4% *versus* 26.3%–33.1% in the “breastfeeding never or <3 months” category). However further multivariate analyses displayed no significant trend.

The proportions in Table 2 are similar (and not significantly different) between males and females although in multivariate analysis (Figure 1), boys were less likely to be breastfed for more than three months.

Variables removed from multivariate analysis were primary caregiver occupational skill level and quintiles of socio economic disadvantage as they were not significantly associated with breastfeeding. Although proportionately more children were breastfed for three months or more when their mothers chewed tobacco during pregnancy (yes = 87%, no = 70%), mothers that did chew tobacco (*n* = 760) were inconsequential compared to those that did not (*n* = 21,300) hence significance was not reached.

## 4. Discussion

The objective of this study was to look at determinants of breastfeeding duration in Aboriginal infants and children in Western Australia, with a particular focus on area of location of residence. We have shown that greater residential isolation, being a non-smoker during pregnancy, higher levels of maternal education, birth weight of 2500 g or more, being female, and having four or more children in the family were protective factors for longer breastfeeding duration in Aboriginal infants and children. 

Comparisons show that in Western Australia, breastfeeding initiation and duration rates are higher in general, than many other Australian states [17]. Aboriginal mothers in Perth, Western Australia were shown to have breastfeeding rates that were on par with non-Aboriginal mothers [18]. In a Melbourne study of Aboriginal infants, at aged three months, only 50% of babies were being breastfed [19]. In a Brisbane based study of Aboriginal people from an urban community, 42% of mothers who commenced breastfeeding were still breastfeeding at three months [20] compared with Western Australian mothers groups where breastfeeding rates at three months or more were 71% [7,21]. The increase in the latter study could be attributed to the inclusion of non-urban Aboriginal groups, as national statistics show that urban Aboriginal populations have lower breastfeeding rates [22,23,24]. 

Even when accounting for confounding measures in our study, 91% of Aboriginal children living in extremely isolated locations were being breastfed for three months or longer. Other recent research also shows that breastfeeding rates in areas of increased levels of relative isolation, where the proportion of Aboriginal people is greater, were higher than for other Australians [23]. Similarly it was found that breastfeeding for 12 months or more proportionately increased with residential isolation, ranging from 27% in the Perth metropolitan area up to almost half (49%) of all Aboriginal children in areas of extreme isolation [7]. Most Australian Aboriginal populations live in urban areas and have introduced Westernised practices such as formula feeding [21]. Historical information shows that traditionally, Aboriginal mothers breastfed for up to four years, and at least the first six months were exclusive breastfeeding [25]. It is thought that the maintenance of a traditional lifestyle is related to the prevalence of breast-feeding in more isolated areas [5]. Coupled with limited access to infant foods, safe water and bottle cleaning facilities, these could be reasons for increased breastfeeding duration in more isolated areas. 

Smoking during pregnancy was shown to be associated with decreased breastfeeding duration in our study. The National Aboriginal and Torres Strait Islander survey (NATSIS) found the proportion of current female smokers was lowest in rural areas and highest in capital cities [26]. This could be related to the differences in availability of tobacco and cigarettes in these locations.

Our research showed that Aboriginal children from families with four or more children were more likely than families with three or less children to be breastfed for three months or more (77.8% *versus* 65.8%). Further analyses showed that these children were 1.28 times more likely to be breastfed for three months or longer. There are difficulties in comparing our results to other studies, as the parameters of variables differ, in that no other study that we are aware of to date, have dichotomized their results as we did. Simard *et al.* [27] reported a longer duration of breastfeeding with increased parity (*p* = 0.012). However, they categorised parity as “0” (*i.e.*, expecting their first child at the beginning of the study), “1” and “≥2” children. In addition, given parity is looking at births and not necessarily number of children, it is difficult to make an accurate comparison between our results and other studies.

Prevalent low birth weight is evidence of the poor nutritional status of Aboriginal infants and possibly mothers [5]. We found that infants with birth weights less than 2500 g were less likely to be breastfed for three months or more. In their review of the literature, Scott and Binns [6] found that infants with low birth weights could have health problems diagnosed at birth that may prevent the early initiation, and subsequent adequate duration, of breastfeeding. Management of health problems may require separation of an infant from their mother which restricts access for breastfeeding. Hospitals with specialised facilities to treat infants can be located in cities. For mothers with low birth weight infants in hospital who are from isolated areas, restricted access could be compounded if they are not able to stay with their infants to breastfeed. Scott and Binns [6] suggested key factors in overcoming breastfeeding impediments, are strong maternal commitment and an encouraging hospital environment.

We note that there was small significant difference in breastfeeding between boys and girls that has not been explored in the empirical literature and is counter to some narratives (that boys are fed for longer in Aboriginal families) [28]. 

### Strengths and Limitations

The study was undertaken with data from a large and representative survey (the WAACHS) of Aboriginal child health, development and education that was conducted using robust and culturally appropriate methods and processes. The use of Aboriginal interviewers, where possible, was a particular strength of the survey design. Other studies have used similar methods to build trust among the study responders, potentially facilitating greater accuracy in responses given [29,30]. In the WAACHS study more than 130 interviewers, 60% being Aboriginal were trained. Seven days of training plus data collection trials saw the numbers drop to 68 interviewers, 25% of whom were Aboriginal. Due to the small Aboriginal population in Western Australia, a consequence of this being that many Aboriginal people know each other, there was a requirement of Aboriginal data collectors to be well qualified and strategically deployed to minimise potential confidentiality issues [21]. 

The survey was specifically designed to produce a sample that could be weighted to estimates that reflect the population of Aboriginal children in Western Australia. Post-stratification weights were applied to the sample to adjust for differential non-response, *i.e.*, to adjust for the modest bias in the sample by child age, household size and region. As a result, the weighted population estimates provides a more robust (unbiased) picture of the group of interest than what could be achieved by analysing sample (unweighted) data. The application of survey weights has been strongly encouraged by the survey Steering Committee and has been consistently applied to all outputs from this source [7,8,9,10,15,21,31].

A potential limitation for the accuracy of WAACHS data used in our study was if maternal recall was biased, in particular by time. The oldest WAACHS children were born in 1982 [7], hence some primary caregivers, at the time of the survey were being asked to recall events from up to 20 years before. Oddy *et al.*, who examined a similar subset of WAACHS children in their study, “looked at the proportion of Aboriginal children ever breastfed, breastfed for six months and breastfed for 12 months of more, by age at time of survey” [21] to determine whether the validity of duration of breastfeeding data was affected by recall bias. There was no change over time of the proportion of breastfeeding per category. They concluded that WAACHS breastfeeding duration data were precisely recalled [21]. This mirrors findings from other studies that maternal recall is a reliable and valid approximation of breastfeeding duration [32]. 

There appears to be a lack of reliable time series data on Aboriginal breastfeeding and therefore it is difficult to ascertain whether breastfeeding rates have changed significantly in the last 10–12 years. While the data source is now over ten years old, they still provide a reliable assessment of the health, social and economic circumstances of Aboriginal children and families as there have been few significant changes in these circumstances across Australia since the data were collected. Certainly, many of the other indicators of wellbeing have remained static and accordingly, the data continue to be a relevant and useful resource for examination of the circumstances of Aboriginal and Torres Strait Islander children. 

Information on drugs used in labour (and on the circumstances of the delivery, more broadly) were not available from the survey and therefore we were not able to account for these potential confounders in the analysis, which we note as a limitation [33]. 

## 5. Conclusions

The objective of this study was to examine determinants of breastfeeding duration in Western Australian Aboriginal children. We have shown there is a strong gradient for breastfeeding duration by remoteness where greater residential isolation is a protective factor. In addition being a non-smoker during pregnancy, higher levels of maternal education, birth weight of 2500 g or more, being female, and having four or more children in the family were protective factors for longer breastfeeding duration. Although this observational, retrospective analysis can limit the conclusions that are drawn, we have substantiated that the methods used lead to accurate maternal recall of breastfeeding duration in the WAACHS data. 

Health promotion programs highlighting the strengths that mothers of Aboriginal children display in longer breastfeeding duration, should be developed. Such programs could simultaneously encourage a reduction in adversely associated factors including maternal smoking and low levels of education, and low birth weight infants. The findings from this study could be used as a starting point for positive reinforcement public health interventions for the benefit of Aboriginal infants and children.
